# From Content Knowledge to Community Change: A Review of Representations of Environmental Health Literacy

**DOI:** 10.3390/ijerph15030466

**Published:** 2018-03-07

**Authors:** Kathleen M. Gray

**Affiliations:** Institute for the Environment, Center for Environmental Health and Susceptibility, University of North Carolina, Chapel Hill, NC 27599-1105, USA; kgray@unc.edu; Tel.: +1-919-966-9799

**Keywords:** environmental health literacy, environmental exposure, environmental literacy, science literacy, self-efficacy, community-based participatory research (CBPR)

## Abstract

Environmental health literacy (EHL) is a relatively new framework for conceptualizing how people understand and use information about potentially harmful environmental exposures and their influence on health. As such, information on the characterization and measurement of EHL is limited. This review provides an overview of EHL as presented in peer-reviewed literature and aggregates studies based on whether they represent individual level EHL or community level EHL or both. A range of assessment tools has been used to measure EHL, with many studies relying on pre-/post-assessment; however, a broader suite of assessment tools may be needed to capture community-wide outcomes. This review also suggests that the definition of EHL should explicitly include community change or collective action as an important longer-term outcome and proposes a refinement of previous representations of EHL as a theoretical framework, to include self-efficacy.

## 1. Introduction

Environmental health literacy (EHL) is an emerging framework that describes a range of knowledge and skills that enable people to make health-protective decisions using available environmental data. At its most basic, EHL has been described as an ability to make connections between environmental exposures and human health [1]. Representations of EHL tend to start with individual understanding of specific risks and then lead to broader understanding, including strategies that empower people to reduce or eliminate environmental exposures that can harm health. The Society for Public Health Education (SOPHE) defined EHL as “the wide range of skills and competencies that people need to seek out, comprehend, evaluate, and use environmental health information to make informed choices, reduce health risks, improve quality of life and protect the environment” [2].

One of the earliest references to EHL in the peer-reviewed literature was from a case study that described the process of producing a community guide to brownfields [3]. The authors, who included literacy experts, environmental scientists, and residents of communities with brownfields, focused on the use of oral and written language to understand basic concepts that are related to the interaction of health and environment and the subsequent use of such information to make decisions that protected against potential hazards. The authors asserted that increased EHL would lead to greater action to protect human and environmental health.

EHL is a natural outgrowth of other literacies, including science literacy, health literacy, and environmental literacy; and these merit brief discussions to contextualize EHL. Since the 1980s, science literacy has been a goal of primary and secondary science education, and it is now a mainstay of discussions of public understanding of science [4]. In 1990, a science-literate person was described as someone who understood the interdependence of science, mathematics, and technology, as well as key scientific concepts and the use of scientific knowledge for individual and societal benefit [5]. In the landmark document *Science for All Americans* [5] environmental issues were highlighted in the introduction, with references to unchecked population growth, acid rain, shrinking tropical rain forests, loss of species diversity, and pollution of the environment mentioned as part of the rationale for fostering science literacy. Despite this early emphasis on societal impacts, efforts to measure science literacy focused on individuals’ understanding of vocabulary and scientific inquiry, resulting in a greater emphasis on individual science literacy than societal [4,6].

Similarly, health literacy focused primarily on individual knowledge and actions, by design, because research has shown that people with low health literacy have difficulty understanding health information, get less preventive care and pay more for their care [7]. Thus, the Institute of Medicine described a health literate individual as someone had the capacity to obtain, understand, and apply information in ways that enabled appropriate health decisions [8] (Executive Summary, p. 2). However, Freedman [9] asserted that focusing on individual capacity and competency ensured that low levels of health literacy were associated with individual deficiencies, rather than socio-cultural dynamics. Instead, he proposed the concept of public health literacy (PHL), which was defined as “the degree to which individuals and groups can obtain, process, understand, evaluate, and act upon information needed to make public health decisions that benefit the community” [9] (p. 448). This concept recognized that individuals exist in environmental and social contexts and assumed that individuals and groups had the power to organize activities to accomplish public health goals through civic engagement.

Along these lines, an environmentally literate person has been defined as someone who works individually and with other people to make decisions to protect the environment, explicitly incorporating a civic engagement component [10]. Despite the focus on collective action, critical commentary has highlighted the “culturally specific” knowledge that is embodied in environmental literacy, noting that it fostered “particular ways of thinking and acting in the world” [11] (p. 39), with a primary focus on White, western values. Given the emphasis on civic engagement, Cole [11] highlighted the importance of addressing how human history has influenced and defined political systems. Further, she argued for examination of biases and pedagogical goals and questioned whose knowledge was included or excluded, a point that is relevant to any of the literacies described herein.

As these examples indicate, over time, the literacy conversation has begun to include sociocultural dimensions, recognizing that individuals acting alone may not be able to influence community-scale and policy issues [4]. This shift is evident in the emerging representations of EHL as a continuum of knowledge, skills, and practice [1,12,13]. Yet, as a new framework, limited information is available on how researchers are representing and measuring EHL. For this reason, this paper summarizes the current peer-reviewed literature to answer two questions: (1) how is EHL characterized in the peer-reviewed literature, and (2) how is EHL measured? This paper also identifies theoretical frameworks that have been applied and considers the extent to which cognitive, affective and behavioral learning has been incorporated into researchers’ representations of EHL. Synthesizing across studies, this review provides an overview of EHL as presented in peer-reviewed literature and suggests a refinement of previous representations of EHL as a theoretical framework.

## 2. Methods

This paper reviews 31 research articles addressing EHL and published in peer-reviewed journals in English, between 2000 and 2017. Because EHL is a relatively new conceptual framework, two research briefs (presenting preliminary results), two case studies, and a commentary are included among the reviewed articles.

### 2.1. Definitions and Search Methods

Several criteria were used to identify relevant studies, with emphasis on the type of environmental exposures addressed and participant understanding of environmental exposures. The articles reviewed in this paper addressed physical, chemical, and biological exposures in immediate or proximate surroundings (e.g., soil, water, air and food) that affected United States residents in their homes, neighborhoods and communities. In many cases, participant understanding of the influence of these exposures on health was explicitly evaluated. Environmental exposure studies were included when researchers actively engaged participants in the process of communicating study results or when participant understanding of results was assessed.

Studies were excluded if they addressed distal concerns (such as climate change) or they were environmental exposure studies with neither engagement of study participants nor an explicit focus on helping participants to understand results. Studies that focused on improving health literacy, when narrowly defined as patient-provider interactions, also were excluded, as were studies of risk perception that primarily examined the cognitive process of risk judgments that people make when they are asked to evaluate environmental activities and technologies.

### 2.2. Screening Methods

**Figure 1:**
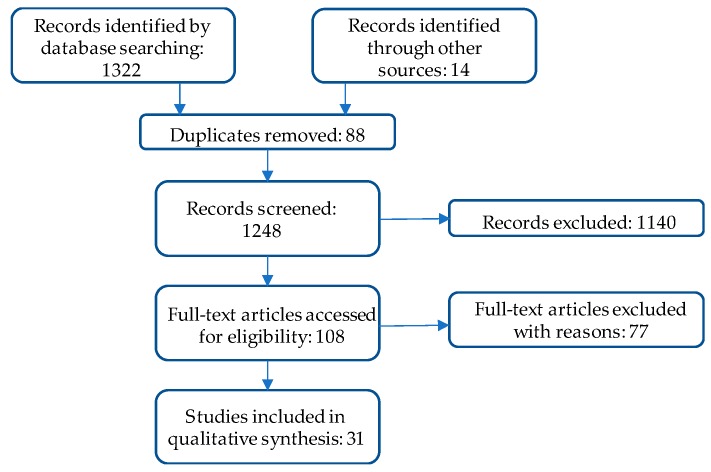
Study selection.

This systematic review was conducted using three databases: Academic Search Complete, ProQuest Environmental Science Collection, and Web of Science. Search terms included: environmental health literacy, environmental health and literacy, and environmental literacy and health education. The three databases returned 1322 results: 162, 1105, and 55, respectively. Of these, 88 duplicate studies were removed. Full text was retrieved for 108 studies, and of those, 75 were rejected (See Figure 1).

In the screening process, abstracts were examined to determine whether articles met the inclusion criteria. For each potentially relevant article, the following questions were asked: Did this study address a physical, chemical or biological exposure? Did this study discuss exposure in relation to health or perceived health risk? Was participant knowledge or understanding of environmental exposures assessed or was participant feedback sought in reporting of results? Reference lists in relevant studies also were scanned for additional studies. For each article that met these criteria, the following information was catalogued: author, year, journal title, environmental exposure, methods, findings, and funding sources. Not all of the studies focused on the same exposure, nor were findings pooled and compared using a common metric.

## 3. Results

As noted above, 31 articles met the inclusion criteria for articles addressing environmental health literacy. Brief summaries are presented in Table 1. The environmental exposures featured in these studies ranged from broadly defined environmental health hazards in communities (e.g., air and water pollution) to specific exposures, such as arsenic in soils and dioxin in water. In addition to arsenic and dioxin, specific toxicants included endocrine disrupting chemicals, environmental tobacco smoke, lead, manganese, mercury, mold, perfluorooctonate (PFOA), pesticides and radon, among others. In terms of trends in publication, these articles were published in 21 different journals, and 20 of the 31 articles were published in 2014 or later, underscoring that EHL is an emerging area.

Looking across studies, approaches to describing and assessing EHL can be grouped by whether the research was focused at individual or community levels or both. Specifically, the studies represented EHL in the following ways:

- Individual-level EHL was described as: (a) understanding the connection between environmental exposures and health; (b) representations of content knowledge, such as a score on a survey of environmental health knowledge or gains in content knowledge demonstrated with pre/post-assessments; and, (c) behavior changes reported in response to environmental exposures.
- EHL that spanned individual and community levels appeared in biomonitoring studies that emphasized “report-back” of individual and community-wide results to participants.
- Community-level EHL was represented as community change or collective action reported in response to environmental exposures.

Below are brief descriptions of the reviewed studies, organized by these categories.

### 3.1. Individual Level EHL: Understanding the Connection between Environmental Exposures and Health

Several studies explored the connections participants made between environmental exposures and their health, with study samples that included students, patient populations, and community residents. Two of these studies focused on endocrine disrupting chemicals (EDCs) and found varying levels of awareness of how exposure to EDCs might affect health. With a sample of 72 female college students, Chan, Chalupka, and Barrett [14] found that participants lacked awareness of potential health effects of environmental toxicants in personal care products (e.g., personal hygiene products, cosmetics, etc.), despite using a high number of them. Participants also reported greater concern about global environmental issues such as air and water pollution than toxicants in personal care products. In the context of prenatal education, Chen et al. [15] assessed how 124 women perceived risks of exposure to EDCs during pregnancy. The researchers found that, despite universal agreement that women wanted healthy babies, they did not connect exposures during pregnancy to future health outcomes for children, leading them to dismiss some claims or to avoid protective actions related to environmental exposures.

Barrett et al. [16] surveyed 894 pregnant women about their attitudes towards environmental chemicals in food and personal care products and their decisions to limit exposure to such chemicals. The authors reported that college-educated participants were more likely to believe that environmental chemicals were dangerous; and, these participants also were more likely to report healthy behavior choices that would limit exposure to such chemicals. In contrast, younger women were more likely to believe that it was impossible to limit exposure to environmental chemicals.

In another study, researchers partnered with a local youth-serving organization to form a Youth Advisory Council, which informed and participated in all the phases of a project that was aimed at assessing youth knowledge of lead poisoning and understanding how they conceptualized and prioritized environmental health concerns [17]. The research team found that youth knowledge was limited and included substantial misinformation. Although the youth were generally aware of lead sources, their knowledge of causal pathways and prevention strategies was limited.

Other studies have shown that participants defined environmental health broadly, connecting many aspects of their communities with potential health outcomes. For example, members of the Confederated Tribes of the Umatilla Indian Reservation identified a broad range of environmental health concerns [19]. The environmental exposures of concern included water pollution from hazardous waste sites and cattle ranching and poor air quality from industrial sources, smoking, and mold. Participants also included toxic chemical exposure from pesticides, a nuclear facility and methamphetamine labs. Further, participants noted that “unhealthy commercial food choices and pollutants in the natural environments” [19] (p. 119) were the driving factors for disease in their community.

White, Hall, and Johnson [20] sought to understand how residents of a public housing development in Chicago understood environmental health hazards. The authors found that when residents defined environmental health risk factors, they included risks from pollutants as well as physical safety concerns from crime and law enforcement interactions. They identified several physical and social hazards as risks to their families and the larger community, with the following being identified as most hazardous: dumping of hazardous waste, landfills, crime, and drugs.

Similarly, in a case study that is focused on documenting how community-based participatory research could inform community organizing, the authors described an academic-community partnership that documented health concerns of a low-income community of color in California, one that was disproportionately exposed to environmental pollution and neighborhood stressors [18]. The authors showed that violence and neighborhood safety were incorporated into residents’ perceptions of environmental health.

### 3.2. Individual Level EHL: Representations of Content Knowledge

Several studies have focused on developing tools to measure EHL and using these tools to define an EHL baseline. For instance, Ratnapradipa, Wodika, Brown, and Preihs [22] refined a previously-developed EHL survey and found that participants had difficulty understanding terminology, underscoring the technical and local nature of some environmental health issues. This study incorporated by reference two earlier versions of this instrument. One was used to survey 395 undergraduate students that were enrolled in introductory health education courses to determine their level of environmental health awareness [45]. Results suggested a limited understanding of environmental health concepts among participants and a lack of protective behaviors when faced with environmental risks. The other was tested with 101 pre-service teachers [46]; and, although about 55% of participants strongly agreed that environmental health issues affected them and their families, results indicated limited environmental health knowledge.

In another assessment of public understanding of environmental health, researchers developed and validated the Environmental Health Engagement Profile [21]. This survey assessed how respondents understood environmental health hazards and associated risks and gathered data on individual and collective responses to risks. This instrument only included environmental exposures with identified health outcomes. Results suggested that, in response to risks, individual actions were more likely than community-level actions.

Researchers also have demonstrated advances in EHL using pre-/post-assessments to document knowledge gains following educational interventions. These studies have occurred in community settings and in formal educational settings.

#### 3.2.1. Community Settings

Following an in-person environmental health intervention, Ramos, He and Ramos [28] assessed the environmental health knowledge of residents of 498 households along the Texas-Mexico border. For an eight-month period, community health workers provided in-person environmental health education for residents in community settings. Using data from pre- and post-surveys with questions about environment, health, community perceptions, and demographics, the authors reported statistically significant improvements in residents’ literacy in the areas of pesticide-related exposures, water-related exposures, and smoking-related diseases. In another study, the authors assessed 20 farmworkers’ knowledge of pesticide safety messages following an hour-long lesson [26]. With pre-/post-assessment data, the authors demonstrated significant increases in participants’ knowledge, and those who had received training within one year of the study also performed better on the assessments than those who had received training two years earlier. Similarly, breast cancer survivors and lay health advisors educated African-American and Latina women in New York city about environmental exposures and breast cancer risk [23]. Following the educational intervention, the participants showed significant knowledge gains on pre/post-tests.

#### 3.2.2. Formal Educational Settings

According to Cohen, Waters, and Brown [24], knowledge gains among middle school students who participated in an environmental health and justice education program demonstrated the potential of such programs to influence students’ EHL and encourage civic engagement in local cleanup decisions. The program was conducted in three settings: during school hours, as an afterschool club activity and as a spring-break camp; and students demonstrated increased content knowledge in all three settings. Learning objectives were assessed through observation of students’ class participation and artifacts (e.g., notebooks) as well as feedback from school personnel.

With K-12 teachers, researchers measured the environmental health knowledge of 35 teachers following their participation in a two-day training on integrated pest management and chemical use reduction [25]. Using, pre- and post-surveys, researchers assessed participant awareness of topics covered, content knowledge, and pedagogical content knowledge associated with liberating structures (a collaboration and conflict resolution approach that was adapted for classroom use). Awareness levels increased across the board; and although content knowledge did not change significantly, pedagogical content knowledge did.

In health care settings, electronic formats have been used to assess EHL. For instance, a questionnaire was used to evaluate the effectiveness of an e-book in developing environmental health competency among clinical professionals [27]. The e-book explored natural and built environments, chemicals, food, and economic and social influences on health. Evaluation questions focused on content knowledge and the potential application of new knowledge, among other items. Citing self-reported knowledge gains among participants as well as high levels of intention to apply new knowledge in practice, the authors asserted that the e-book provided an accessible method of increasing EHL. In another study, which was aimed at testing the effectiveness of electronic kiosks for communicating environmental health information, results of pre-/post-tests showed that participants chose significantly more correct answers on post-tests [29]. The authors also found that the kiosks were an acceptable strategy for sharing information on environmental exposures.

### 3.3. Individual Level EHL: Behavior Change in Response to Environmental Exposures

Several studies have reported individual behavior changes in response to environmental health education. For example, Korfmacher and Kuholski [32] demonstrated the impact of a community-wide initiative to educate residents about lead, asthma and other environmental health hazards in homes. One component of their educational initiative was a physical *Healthy Home*, with low-literacy materials and hands-on activities displayed throughout the home providing examples of how to reduce environmental health hazards. Following a home tour, the visitors identified actions that they intended to take to reduce hazards in their own homes. Using phone and email follow-up (with over a third of the initial sample of 337 visitors, *n* = 119), the authors documented that almost all of the respondents had completed or partially completed the identified actions.

In another study, a risk communication intervention was developed, implemented and evaluated in a low-income, predominantly African American public housing community in a southeastern state [31]. This study explicitly referenced social cognitive theory [47] as the basis for design of key aspects of the intervention, including use of race-specific role models as educators and participatory input in the content, specifically related to fishing behaviors. In-person surveys were conducted with 23 community residents at baseline and three months post-intervention to assess the changes in knowledge and behaviors. Findings showed knowledge gains, and participants reported behavior changes related to fish preparation and reduced consumption of larger fish, fish eggs and internal organs, as well as reduced consumption of fish by pregnant women and children.

More recently, New Hampshire environmental and health agencies partnered with a local conservation organization and researchers to raise awareness of potential contaminants in well water and encourage water testing [34]. The program actively engaged the local community and was designed to address identified barriers to water testing, such as limited access to test kits, sparse test facilities in rural areas, and misperceptions about contaminated water (i.e., that it tastes or smells bad). As a result, well testing rates increased, demonstrating that a participatory program that reduced barriers to well testing could increase the likelihood of protective behaviors.

In a community with a large coal ash storage site, researchers [36] surveyed 231 residents who lived close to the facilities to learn about the potential exposure-reducing behaviors. The researchers found that residents who made connections between coal ash exposure and poor health were more likely to engage in exposure-reducing behaviors. This finding was tempered by feelings among some participants of helplessness regarding their ability to avoid the pollution.

#### The Role of Community Health Workers

A subset of studies has demonstrated the value of community health workers and home-visiting professionals in environmental health interventions, and these studies explicitly incorporated self-efficacy into study design. Working with 150 urban families in a northeastern state, Mankikar, Campbell, and Greenberg [33] analyzed the influence of a home-based environmental health intervention on knowledge of environmental health hazards, presence of hazards in homes, and medical visits for children with asthma. Two home visits were conducted by community health workers and included a home assessment (with pre-/post-assessments), environmental education, and distribution of supplies. Following the intervention, the authors reported that participants’ knowledge of environmental health hazards had increased, home hazards were reduced, and participants reported fewer medical visits.

Similarly, in 235 homes in the northwest, Butterfield et al. [30] assessed the influence of a home intervention on the presence of environmental health hazards (e.g., lead, radon, cigarette smoke). The intervention, which was led by public health nurses, included the collection of environmental samples and biological samples from children living in the homes. The nurses shared sample results using materials tailored for each participant. Instead of focusing on health outcomes, these researchers assessed the impact on parents’ sense of self-efficacy and adoption of risk-reducing behaviors and reported increases in both outcomes.

In another study relying on community health workers, Quandt et al. [35] prepared lay health advisors (promotoras) to educate families about pesticide exposure. The promotoras delivered a six-lesson, in-home, culturally appropriate curriculum to 610 members of farmworker families. Using pre- and post-assessment, significant improvements in knowledge were reported for all six lessons. Additionally, significant improvements were reported in practices that were related to residential pest control and para-occupational exposure (i.e., exposure of people who live with occupationally exposed workers to substances that have been carried home from the work place). Quandt et al. asserted that these findings showed that lay health advisors with limited training could impact family knowledge and practices.

### 3.4. Spanning Individual and Community-Level EHL: Participation in Report-Back Studies

Biomonitoring studies coupled with extensive report-back protocols have shown how study participants improve their understanding of environmental exposures and use new knowledge to inform action, both at individual and community levels. For instance, Haynes et al. [39] described a community-engaged approach to sharing data associated with an environmental exposure study. Following the discovery of airborne manganese exceeding reference concentrations in a Midwestern community, 64 households (with 104 children) provided samples of children’s blood and hair, which were analyzed for metals. Data sharing strategies were developed using an iterative process between academic and community partners; and, this process informed the decision to communicate results graphically, in community meeting, and individual letters to each family. A post-meeting survey indicated that all of the participants understood their results.

Similarly, Ramirez-Andreotta et al. [41] sought to determine whether parents of children who had participated in a toxic metals exposure study understood their children’s results. Findings indicated that they not only understood the results, but also used the data to ask new questions and reduce their family’s health risks, leading the authors to assert that the study advanced participants’ EHL.

In a multi-year study of young women’s exposure to endocrine disrupting compounds (EDCs) in personal care products, Madrigal et al. [40] reported increased EHL among youth participants. Participating youth learned to measure their EDC exposure and assisted researchers in developing educational materials for other youth. They also helped to publicize study findings and develop relevant advocacy activities. Researchers used written reflections, end-of-event surveys, interviews, and observation (by both adult and youth participants) to assess participants’ EHL as well as their leadership skills, career orientation and self-esteem. One EHL metric that was highlighted by Madrigal et al. was participants’ ability to discuss relevant content fluently and with professionalism with varied audiences (from families to legislators).

In the Northern CA Household Exposure Study, researchers analyzed the impact of community context and report-back processes on participants’ perceptions of study results [37]. Two communities participated, one on a refinery fence line and the other in a wealthy, rural area. Results showed that residents in the fence-line community expected pollution exposure from industry but not from household products; and, the wealthy community was particularly surprised to learn of contamination from indoor sources. Although residents in both of the communities had a general awareness of the connection between environmental exposures and health, they deepened their knowledge by learning about the effects of cumulative exposure to multiple chemicals. This new knowledge informed personal choices and collective action to address exposures from outdoor industrial sources, specifically changes to oil refinery permitting processes. The authors argued that approaches that actively engaged in the public in science, such as community-based participatory research, increased understanding and literacy among lay people and scientists.

In a companion study, the researchers used the results of an extensive self-evaluation of the Household Exposure Study to assert that study participants had increased their EHL while supporting community empowerment activity and generating policy results to protect public health [38]. Evaluation questions included whether scientific results led to action and whether research practices contributed to community education or engagement, among others. Data sources included written evaluations and observer notes from community meetings, interviews with participants, notes from community advisory board meetings, and other documentation of study outcomes.

### 3.5. Community Level EHL: Community Changes or Collective Action in Response to Environmental Exposure

Researchers also have represented changes in EHL using evidence of community change or specific actions that were taken by study participants in response to knowledge gains. Ramirez-Andreotta et al. [44] measured EHL by analyzing the changes over time in community concerns related to a mining waste site. They analyzed publicly available data sources associated with the waste site over a five-year period, documenting a shift in community inquiries, from site-specific to focusing on exposure assessment, and potential influences of the site on local water supplies. The authors asserted that, in addition to representing changes in EHL, documentation of community inquires also enabled an assessment of whether community concerns were adequately addressed.

In a community-based participatory research study focused on the potential health impacts of contamination of public water supply wells, researchers found perfluorooctanoic acid (PFOA) in participants’ blood at levels that were ~80 times higher than in the general population [42]. This study relied on an active Community Advisory Committee to provide input into research questions and study design, development of questionnaires, and communication of results. The research team also helped residents to develop response strategies. One outcome was that a polluting industry provided bottled water (accepted by more than 75% of residents) until the local water utility could upgrade its filtration system.

In another study, researchers partnered with residents of a southwestern community with arsenic contamination to determine the extent of arsenic uptake in garden vegetables [43]. They also sought to characterize the potential risks to residents from gardening and consuming vegetables from their gardens. During the study, individual learning, programmatic outcomes, and community-level outcomes were documented. Similar to the report-back studies described above, the results showed that participants understood their data and made changes to reduce their arsenic exposure. At the community-level, the authors reported that participants became more active in other local environmental issues, including the contamination of the public water supply.

## 4. Discussion

Several studies included in this review explored the extent to which participant understanding of potential health risks from environmental exposures could be represented as a form of EHL. These studies had mixed results, in that some showed that participants connected environmental exposures and poor health outcomes [16,17], demonstrating at least basic levels of literacy, while others did not [14]. In those studies where participants made connections or appeared to understand that environmental exposures could influence health, they still expressed misinformation or appeared to be lacking essential information to identify strategies for reducing exposure [17,22]. Participants also defined environmental exposures more broadly than they traditionally have been defined in environmental health sciences, for instance, including crime and violence in their definitions [18,20]. In terms of behaviors in response to environmental exposure or education about such exposures, some studies reported individual behavior changes [31,33,35], while others reported community-wide impacts [42,43].

Despite heterogeneity among methods, several theoretical frameworks were employed in multiple studies, including: community-based participatory research (CBPR), the Health Belief Model, and social-cognitive theory. The most commonly applied theoretical framework was CBPR [17,18,24,39,40,42,43,44]; and, many studies referenced the adoption of the key principles of CBPR [48], even when the framework was not rigorously applied. This emphasis underscores the role of social interaction in learning and constructing meaning [49], and suggests that movement along the continuum of EHL may be inherently tied to active engagement of participants throughout the research process, especially in communities directly impacted by environmental contamination. Interestingly, several studies explicitly incorporated self-efficacy, with references to social-cognitive theory [47] and the Health Belief Model [50]. These tended to include educational interventions designed for use with specific audiences (e.g., fishermen, parents, pesticide educators), and in some cases, the researchers reported improvements in self-efficacy as representing EHL. Additionally, most studies included in this review occurred in free-choice learning contexts (i.e., settings outside of formal classrooms that enable self-motivated learning), providing a range of examples of how researchers can characterize and measure EHL in these contexts.

To date, just a few instruments have been developed and validated specifically for use in assessing EHL [22,33], while others have been adapted from existing instruments [17,31]. Ratnapradipa et al. [22] reported that the local and technical nature of environmental health issues complicated the development of a broad instrument, emphasizing the contextual nature of environmental health learning.

Surveys, focus groups and interviews have been used to understand whether and how specific populations understood the influence of environmental exposures on health [14,15,20,36]. Gains in content knowledge using a pre-/post-test design were another method of demonstrating changes in EHL [24,25,28]. Pre-/post-assessments also were used to document behavior changes—planned or actual—in response to environmental exposures [23,25,26,27,28,29]. Dixon et al. [21] found that individual actions were more likely than community-level actions as a response to information about a harmful environmental exposure.

Biomonitoring and report-back studies actively engaged participants throughout study design, implementation and sharing of results, and most reported increased EHL as a result [37,40,41,42,43,45]. Among the studies that reported community-level outcomes, these outcomes included provision of alternate community water supplies [42] and greater involvement in other local environmental issues [43]. Typically, these studies applied CBPR as a theoretical framework and provided insight into the resources, time and expertise needed to accomplish such outcomes. Assessment of other CBPR efforts, not limited to environmental health, suggests a variety of metrics that could be used to measure the impact of increases in EHL, such as changes to human, social and financial resources and increases in perceived control or community cohesion, to name a few [51].

Taken together, these articles have several implications for how EHL is characterized and measured. First, while recognizing that EHL develops progressively, it seems important that the definition of EHL explicitly identify collective action or civic engagement as a desired longer-term outcome, just as environmental literacy does [10]. In many cases, environmental exposures are not under the control of an individual, meaning that community-level action would be required to significantly reduce or eliminate the exposure. In these cases, sustained engagement and efforts that span individuals and communities will be required. As noted above, if collective action or community change is a desired outcome, a broader suite of assessment tools (beyond pre-/post-assessments) is needed, including metrics that indicate actions taken to reduce exposures, such as reported in several of the CBPR studies included in this review [40,41,42,45].

**Figure 2:**
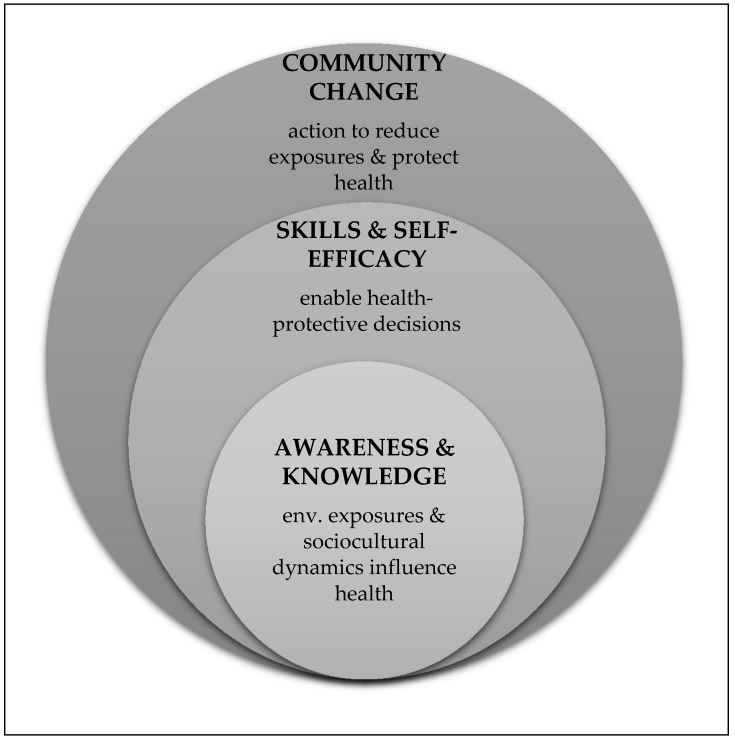
Three dimensions of environmental health literacy (EHL).

This review suggests that awareness and knowledge are an important starting point for EHL, and when combined with information-seeking and decision-making skills and self-efficacy for specific behaviors, such knowledge can inform collective action and community change. Thus, EHL can be conceived of as having three dimensions, each of which builds upon the next (Figure 2):

1. Awareness and understanding: This dimension incorporates the broad recognition that environmental exposures and socio-cultural dynamics influence health. Such awareness may occur in the context of a specific environmental exposure (such as arsenic in groundwater); and, an individual may have varying levels of awareness across different exposures, as represented by Finn and O’Fallon [1]. Alternately, this awareness may reflect a more general understanding that environmental exposures interact with biological processes to cause negative health outcomes. Presumably, either type of understanding would incorporate some recognition of the limits of current science and uncertainty in scientific research.
2. Skills that enable health protective decision-making and self-efficacy associated with those skills: This dimension incorporates social cognitive theory by focusing on an individual’s self-efficacy for reducing harmful environmental exposures as well as beliefs about her/his potential to influence a specific outcome. Mastery of relevant skills is an important component of this dimension; and such skills may be general in nature (e.g., the ability to find and understand scientific information or the ability to participate in community decision-making) or exposure-specific (e.g., the ability to take steps to reduce environmental asthma triggers in a home).
3. Community change or collective action to reduce or remove harmful environmental exposures: In this dimension, both individuals and groups apply their knowledge and skills, in the context of self-efficacy for the desired behavior change, to reduce harmful environmental exposures and improve health. This review suggests that community change outcomes may require an overarching theoretical framework that engages participants throughout the research process and taps into their funds of knowledge and self-efficacy.

This representation of EHL incorporates content knowledge, a core element of science literacy, while also integrating the community-scale and action-oriented lenses of environmental literacy and public health literacy. Additionally, it recognizes the importance of community action in reducing or eliminating some harmful environmental exposures. Although this representation does not address how cultural context influences each stage of EHL, several of the reviewed studies provide relevant examples [18,19,20,31,35,37]. Going forward, explicit consideration of the role of cultural context is needed to ensure that the culturally specific knowledge of non-dominant groups is incorporated as measurement baselines are established for EHL, and as skills-based training and exposure reduction strategies are developed.

## 5. Conclusions

This review of 31 articles underscores the progressive nature of EHL, starting with understanding environmental health sciences broadly, as well as understanding that specific environmental exposures can harm health in known and unknown ways, and leading to informed decisions and actions to reduce or eliminate harmful exposures. This review highlights a range of ways that EHL has been measured and points to the need for qualitative measures and varied approaches to demonstrating changes in EHL.

Further, this review suggests that individuals and communities may move along a continuum of EHL, which connects understanding of how environmental exposures influence health with individual and community action, by explicitly incorporating elements of self-efficacy and development of skills that enable health protective decisions. Building on the findings of the efficacy-based studies that are reviewed here, as well as the principles of CBPR, has the potential to inform environmental health literacy efforts so they may more effectively facilitate movement along this continuum and prompt individual and community-level actions to protect health.

## Figures and Tables

**Table 1:** Articles that Inform the Characterization and Measurement of Environmental Health Literacy (EHL).

Author & Date	Journal	Study Participants	Environmental Exposure	Methods	Theoretical Framework	Funding Source
**Individual Understanding of Connection between Environmental Exposures and Health**						
Barrett et al. (2014) [16]	*Eur. J. Obs. Gynecol. Reprod. Biol.*	894 pregnant women	Environmental chemicals in personal care products	Questionnaires	Not specified	National Institute of Environmental Health Sciences (NIEHS)
Bogar, S., Szabo, A., Woodruff, S., & Johnson, S. (2017) [17]	*J. Community Health*	169 urban youth	Lead poisoning and community-identified environmental health (EH) issues	Survey, focus groups	Community-based participatory research (CBPR)	Medical College of Wisconsin, Purple Door Ice Cream
Chan, L.M., Chalupka, S.M., & Barrett, R. (2015) [14]	*Workplace Health Saf.*	72 female college students	Endocrine disrupting chemicals (EDCs) in personal care products	Survey	Integrated Model for Environmental Health Research	Worcester State Foundation
Chen, S., Barrett, E.S., Velez, M., Conn, K., Heinert, S., & Qiu, X. (2014) [15]	*Policy Futures Educ.*	124 women	EDC exposure during pregnancy	Semi-structured interviews, survey	Health Belief Model	NIEHS
Cohen, A.K., Lopez, A., Malloy, N., & Morello-Frosch, R. (2014) [18]	*Environ. Justice*	188 residents of environmental justice (EJ) community in California	Local environmental pollution (e.g., petrochemical industry) & neighborhood stressors	Community health survey	CBPR	Avon Foundation
Schure, M.B. et al. (2013) [19]	*Environ. Justice*	27 members of the Confederated Tribes of the Umatilla Indian Reservation	Air pollution, water pollution, toxic chemicals	Focus group	Not specified	NIEHS
White, B.M., Hall, E.S., & Johnson, C. (2014) [20]	*J. Environ. Health*	42 adult residents of Chicago public housing	Environmental hazards in community (air, land & water)	Focus groups, surveys	Not specified	Univ. of Minnesota School of Public Health
**Representation of Content Knowledge**						
***Scores and Other Representations of Environmental Health Knowledge***						
Dixon, J.K., Hendrickson, K.C., Ercolano, E., Quackenbush, R., & Dixon, J.P. (2009) [21]	*Public Health Nurs.*	433 urban residents in Northeastern state	Varied sources of pollution linked with health effects	Focus groups, in-person and phone interviews	Not specified	NIEHS
Ratnapradipa, D., Middleton, W.K., Wodika, A.B., Brown, S., & Priehs, K. (2015) [22]	*J. Environ. Health*	32 individuals in 4 states	Range of exposures including air, water, radiation, waste	Focus groups	Not specified	Southern Illinois University
***Knowledge Gains on Pre/Post-Assessments***						
Brenner, B., Evans, S., Miller, K., Weinberg, L., Rothenberg, A., Martinez, C., & Jandorf, L. (2015) [23]	*Environ. Justice*	12 volunteer educators, 103 workshop participants	EDCs in personal care products and chemicals in cleaners, plastics and pesticides	Focus group, post-workshop evaluation	Not specified	National Cancer Institute (NCI), NIEHS
Cohen, A.K., Waters, A., & Brown, P. (2012) [24]	*Environ. Justice*	Middle school students	Dioxin	Pre/post-surveys, student reflections, writing & discussion	CBPR principles, EJ	NIEHS, Brown Univ. Teaching & Research Award
Ferguson, A., Kavouras, I., Ulmer, R., Harris, K., Helm, R., & Bursac, Z. (2014) [25]	*J. Community Med. Health Educ.*	35 teachers	Pesticides and other chemicals used in homes	Pre/post-surveys	Cooperative learning	Not specified
LePrevost, C.E., Storm, J.F., Asuaje, C.R., Arellano, C., & Cope, W.G. (2014) [26]	*J. Agromed.*	20 farmworkers	Pesticides	Pre/post-assessments	Not specified	NC Dept. of Agriculture & Consumer Services
Miller, M.D., Valenti, M., Schettler, T., & Tencza, B. (2016) [27]	*Environ. Health Perspect.*	304 health professionals	Factors in natural, built chemical, food, economic & social environments	Embedded questions in online course	Not specified	Agency for Toxic Substances and Disease Registry (ATSDR), US Environmental Protection Agency (USEPA)
Ramos, I.N., He, Q., & Ramos, K.S. (2012) [28]	*Environ. Justice*	498 households in community on Texas-Mexico border	Pesticides, environmental tobacco smoke (ETS), water pollution	Pre/post surveys administered in-person	Not specified	NIEHS
Rosas, L.G., Trujillo, C., Camacho, J., Madrigal, D., Bradman, A., & Eskenazi, B. (2014) [29]	*Patient Educ. Couns.*	152 pregnant, Spanish-speaking patients	Pesticides, metals, toxic household products, ETS, allergens, indoor & outdoor air pollution	Electronic waiting room kiosk, pre/post-questionnaires	Not specified	California Wellness Foundation, NIEHS, USEPA
**Individual Behavior Change in Response to Environmental Exposure**						
Butterfield, P.G., Hill, W., Postma, J., Butterfield, P.W., & Odom-Mayon, T. (2011) [30]	*Am. J. Public Health*	235 families in rural areas of two Northwestern states	EH hazards in homes (carbon monoxide (CO), drinking water contaminants, mold, radon)	Pre/post surveys	Translational environmental research in rural areas (TERRA), Social Cognitive Theory, Weinstein’s Precaution Adoption Model	National Institute of Nursing Research (NINR)
Derrick, C.G., Miller, J.S., & Andrews, J.M. (2008) [31]	*Public Health Nurs.*	23 African-American subsistence anglers residing in public housing	Mercury in fish	Pre/post-surveys	Social cognitive theory	Sigma Theta Tau
Korfmacher, K.S. & Kuholski, K. (2008) [32]	*Environ. Pract.*	32 visitors to healthy home exhibit	EH hazards in homes (asbestos, CO, ETS, lead, mold, pesticides, radon)	Written surveys on-site, follow-up interviews	Not specified	NIEHS
Mankikar, D., Campbell, C. & Greenberg, R. (2016) [33]	*Int. J. Environ. Res. Public Health*	150 families participating in healthy homes program	Environmental health hazards in homes (CO, lead, mold, pests)	Pre/post-questionnaires, observations	Not specified	Health Resources & Services Admin. Block Grant
Paul, M.P., Rigrod, P., Wingate, S., & Borsuk, M.E. (2015) [34]	*J. Environ. Health*	285 well owners	Arsenic in well water	Number of wells sampled	Not specified	NIEHS
Quandt et al. (2013) [35]	*Health Promot. Pract.*	610 family members of farmworkers	Pesticides	Pre/post-questionnaires	Health Belief Model	National Institute of Occupational Safety and Health (NIOSH)
Zierold, K.M., Sears C.G., & Brock, G.N. (2016) [36]	*Health Educ. Behav.*	257 residents of community with large coal ash storage site	Coal ash (particulate matter, metals)	Focus groups, survey	Not specified	None
**Participation in Report-Back Studies**						
Adams, C. et al. (2011) [37]	*J. Health Soc. Behav.*	50 residents of two California communities, one bordering an oil refinery, one comparison	EDCs, pollutants associated with oil refining	Interviews, individual & community meetings	Exposure experience, health social movements, public engagement with science	Not specified
Brown, P., Brody, J.G., Morello-Frosch, R., Tovar, J., Zota, A.R., & Rudel, R.A. (2012) [38]	*Environ. Health Perspect.*	50 residents of two California communities, one bordering an oil refinery, one comparison	EDCs, polybrominated diphenyl ethers (PBDEs)	Multi-faceted evaluation of ongoing CBPR project	CBPR	NIEHS, National Science Foundation (NSF)
Haynes et al. (2016) [39]	*Environ. Health Perspect.*	30 participants in exposure study	Airborne manganese	Community input, surveys	Not specified	NIEHS, NINR
Madrigal, D.S. et al. (2016) [40]	*Int. Q. Community Health Educ.*	15 members of Youth Community Council	EDCs in cosmetics	Written reflections, questionnaires, participant observation	EHL, CBPR	CA Breast Cancer Research Program, NIEHS, USEPA
Ramirez-Andreotta, M.D., Brody, J.G., Lothrop, N., Loh, M., Beamer, P.I., & Brown, P. (2016) [41]	*Int. J. Environ. Res. Public Health*	17 parents who participated in MESH study	Arsenic	Interviews	EHL, EJ, contextual model of learning	NIEHS
**Community Change or Collective Action in Response to an Environmental Exposure**						
Emmett, E.A., Zhang, H., Shofer, F.S., Rodway, N., Desai, C., Freeman, D., & Hufford, M. (2009) [42]	*J. Occup. Environ. Med.*	Residents of rural Appalachian town in Ohio	Perfluorooctanoate (PFOA)	Participatory research design, community meetings, follow-up surveys	CBPR, EJ	NIEHS
Ramirez-Andreotta, M.D., Brusseau, M.L., Artiola, J., Maier, R.M., & Gandolfi, A.J. (2015) [43]	*Int. Public Health J.*	18 participants in Gardenroots training	Arsenic	Survey	CBPR, public participation in scientific research (PPSR)	NASA, NIEHS, USEPA, Alfred P Sloan Foundation, Univ. of Arizona
Ramirez-Andreotta, M.D., Lothrop, N., Wilkinson, S.T., Root, R.A., Artiola, J.F., Klimecki, W., & Loh, M. (2016) [44]	*J. Environ. Stud. Sci.*	Residents of Dewey-Humboldt, Arizona who participated in community involvement (CI) activities	Arsenic	Review of interviews, community meetings and other CI activities	CBPR, EHL, EJ, risk communication,	NIEHS
